# A High-Affinity Nanobody Selectively Recognizing KPC-2/KPC-3: Biochemical and Structural Insights

**DOI:** 10.3390/biom16030369

**Published:** 2026-02-28

**Authors:** Emna Hamdi, Oussema Khamessi, Alessandra Piccirilli, Sayda Dhaouadi, Sinda Zarrouk, Fabrizia Brisdelli, Hafedh Dabbek, Mohamed Hedi Saihi, Balkiss Bouhaouala-Zahar, Rahma Ben Abderrazek, Mariagrazia Perilli

**Affiliations:** 1 Laboratoire Des Biomolécules, Venins et Applications Théranostiques, Institut Pasteur Tunis, Université Tunis El Manar, B.PN°93, 13 Place Pasteur, Tunis 1002, Tunisia; emna.hamdi@etudiant-fst.utm.tn (E.H.); sayda.dhaouadi@pasteur.utm.tn (S.D.); balkiss.bouhaouala@fmt.utm.tn (B.B.-Z.); 2 Dipartimento di Science Cliniche Applicate e Biotecnologiche, Università degli Studi dell’Aquila, Via Veteoio Coppito, 67100 L’Aquila, Italy; alessandra.piccirilli@univaq.it (A.P.); fabrizia.brisdelli@univaq.it (F.B.); mariagrazia.perilli@univaq.it (M.P.); 3 Laboratoire de Bioinforamtiques, Biomathématiques et Biostatistiques, Institut Pasteur Tunis, University of Tunis El Manar, B.PN°93, 13 Place Pasteur, Tunis 1002, Tunisia; oussama.khamessi@isbt.uma.tn; 4 Institut Supérieur de Biotechnologie, BiotechPôlet, Université de Manouba, B.P N°66, Sidi Thabet, Ariana 2020, Tunisia; 5 IPTOMICS Platform, Institut Pasteur Tunis, University of Tunis El Manar, B.PN°93, 13 Place Pasteur, Tunis 1002, Tunisia; sinda.zarrouk@pasteur.tn; 6 Commissariat Régional au Développement Agricol, Rue Slah Ben Youssef, Kebili 4200, Tunisia; kamelsdabbek@gmail.com (H.D.);; 7 Medicine Faculty of Tunis, University of Tunis Manar, Rue Djebel Lakhdhar, B.PN°93, La Rabta 1007, Tunisia

**Keywords:** carbapenem resistance, KPC-3, KPC-2, β-lactamases, nanobodies (VHHs), β-lactamase detection

## Abstract

Carbapenemase-producing bacteria, particularly those expressing the KPC-3 variant, pose a critical global health threat due to their resistance to nearly all β-lactam antibiotics, including carbapenems. Rapid and reliable detection tools are urgently needed to improve infection control and guide patient management. Nanobodies (VHHs) present a promising alternative to conventional antibodies thanks to their high stability, small size, and capacity to access cryptic epitopes. Here, we report the generation and characterization of a nanobody specifically targeting KPC-3. An immune VHH phage display library was constructed, with over 90% of clones containing correctly sized inserts. After three rounds of biopanning, high-specificity binders were identified by ELISA screening. Sequencing identified a nanobody with hallmark VHH features, which was expressed and validated by ELISA and Western blot. Although kinetic assays showed no inhibition of KPC-3 enzymatic activity, interestingly, the nanobody demonstrated high-binding recognition of both KPC-2 and KPC-3 in periplasmic extracts from clinical strains. Structural modeling further supported these results, highlighting favorable interaction surfaces. This study provides the first evidence of a nanobody raised against KPC-3 that recognizes a conserved epitope shared by KPC-3 and KPC-2, underscoring its promising use as a molecular tool for detecting KPC variants and establishing a basis for future affinity maturation toward therapeutic applications.

## 1. Introduction

Multidrug-resistant bacterial infections are a leading cause of morbidity and mortality worldwide, posing an urgent global health challenge [1]. Among frontline treatments, β-lactam antibiotics are widely used due to their broad-spectrum activity and low toxicity [2]. However, their extensive use has accelerated the emergence of resistant pathogens, which evade antimicrobial action through diverse mechanisms [3]. In Gram-negative bacteria, β-lactamase enzymes, which hydrolyze the β-lactam ring essential for antibiotic activity, constitute the predominant mechanism of resistance [4]. Enterobacterales are the primary reservoir of β-lactamases. According to the Ambler classification, β-lactamases are divided into four classes: A, C, and D serine β-lactamases (SBLs), and B metallo-β-lactamases (MBLs) [5]. Within this classification, KPC enzymes, class A carbapenemases, are of particular concern because of their ability to hydrolyze nearly all β-lactams, including carbapenems. Its dissemination has been associated with outbreaks in hospital settings, leading to severe therapeutic failures and high mortality rates due to the global dissemination of multidrug-resistant pathogens [6]. KPC-2 and KPC-3 are the most widespread KPC variants and differ by a single amino acid at position 274, where KPC-3 carries a tyrosine instead of the histidine found in KPC-2 [7]. The structure of KPC is typical of class A β-lactamases, consisting of one α-helical subdomain and one β-sheet subdomain flanked by α-helices. The cleft between these subdomains forms the active site, which includes the four highly conserved motifs and the catalytic serine S70 [8].

β-lactamase inhibitors remain a cornerstone in the fight against β-lactamase–mediated resistance. For KPC-3, inhibitors such as avibactam or vaborbactam have initially proven effective. However, their long-term efficacy is increasingly compromised, since single amino acid substitutions in KPC-3 can drastically reduce inhibitor binding, giving rise to resistant variants and progressively evade existing inhibitors [9,10]. This rapid evolutionary adaptability underscores the urgent need for novel approaches that combine robust detection with epidemiological surveillance.

Current diagnostic strategies for detecting KPC-producing strains rely mainly on molecular approaches, including conventional PCR, multiplex PCR and sequencing-based assays. Although highly sensitive for gene detection, these methods face a significant diagnostic gap as they do not provide information on protein expression, enzyme abundance, or catalytic activity, and therefore do not always correlate with the resistance phenotype. In addition, sequencing-based strategies remain costly and time-consuming, and many tests still require bacterial culture, delaying clinical decision-making. These limitations highlight the need for complementary tools that directly detect KPC enzymes at the protein level, capable of direct, rapid, and robust protein-level detection.

Therefore, integrating innovative strategies with rapid KPC- variant β-lactamases detection tools is essential to sustain effective therapy and strengthen infection control in clinical practice [11].

Among the various detection platforms, Nanobodies (Nbs) represent an extremely versatile scaffold for developing a theragnostic tool due to their unique biochemical and biophysical properties. With a molecular weight of ~15 kDa and compact dimensions (~2.5 × 4 nm), Nbs exhibit excellent solubility, thermal and chemical stability, and resistance to aggregation, making them suitable for large-scale manufacturing and deployment in various clinical settings, including resource-limited environments. Their high sequence similarity with human VH (VH3 gene family) contributes to their low immunogenicity [12]. Unlike conventional antibodies or inhibitors, Nbs can recognize cryptic or conformational epitopes and resist escape mutations thanks to their extensive and adaptable binding interfaces [13]. These properties enable highly sensitive detection and potential inhibition of β-lactamases [13,14,15]. Indeed, previous studies have demonstrated the potent activity of Nbs against diverse β-lactamases, highlighting their promise as both diagnostic and therapeutic tools [16,17,18,19]. Building on our team’s previous success in generating Nbs against NDM-1 [16], we specifically aimed to develop a KPC-3-targeting Nb capable of precise recognition in both purified and periplasmic protein contexts. We constructed an immune VHH phage display library, selected specific binders via biopanning and ELISA, and characterized the selected Nb using Western blot, kinetic assays, and structural analyses. Our findings demonstrate high specificity for KPC-2/KPC-3, supporting its potential as a rapid diagnostic tool and as a scaffold for future therapeutic applications, addressing a critical unmet need in the fight against multidrug-resistant bacterial infections.

## 2. Materials and Methods

### 2.1. VHH Library Construction

The VHH library was generated following established protocols [20,21]. All procedures concerning the dromedary immunization protocols were approved by the Ethical Committee of the Pasteur Institute of Tunis (acceptance reference: 46/19, approval date: 9 July 2019) and were conducted in accordance with institutional guidelines for animal welfare. The dromedary belonged to a local farmer, who gave consent for its participation under the supervision of a veterinarian from the CRDA of Médenine. A dromedary (*Camelus dromedarius*) was immunized with increasing concentrations of purified recombinant KPC-3 enzyme. Peripheral blood samples were collected at defined intervals in anticoagulant tubes, enabling monitoring of the immune response throughout the immunization process. The blood collected three days after the last injection was used to isolate lymphocytes via Ficoll gradient. Total RNA was extracted from the lymphocytes, and 50 µg was reverse-transcribed into cDNA, which served as the template for a two-step PCR amplification of VHH-encoding domains. The first PCR used primers CALL001 (forward, 5′-GTCCTGGCTGCTCTTCTACAAGG-3′) and CALL002 (reverse, 5′-GGTACGTGCTGTTGAACTGTTCC-3′), which anneal to the leader sequence in FR1 at the 5′ end and the CH2 constant region at the 3′ end, respectively. This step amplified both the VHH–Hinge–CH2 domains from heavy-chain antibodies and the VH–Hinge–CH1–CH2 domains from conventional IgGs. The resulting PCR product (~600 bp) corresponding to VHH-Hinge-CH2 domains was excised from agarose gel, and purified using the QIAGEN DNA Extraction Kit (Cat. No. 28704, Qiagen, Hilden, Germany). A second PCR was performed using A6E (5′-GATGTGCAGCTGCAGGAGTCTGGRGGAGG-3′) and PMCF (5′-CTAGTGCGGCCGCTGAGGAGACGGTGACCTGGGT-3′) primers to reamplify the VHH genes while introducing PstI and NotI restriction sites at their 5′ and 3′ ends. Both the amplified VHH genes and the vector were subsequently digested with PstI and NotI restriction enzymes (R3140T and R3189M, New England Biolabs, Hitchin, UK), and ligated using T4 DNA Ligase (15224-041, Invitrogen, Carlsbad, CA, USA). The ligation products were then transformed into *E. coli* TG1 cells, and (TG1), and plated on Petri dishes containing LB-agar with 100 µg/mL ampicillin. Successful cloning of VHH inserts into the pMECS vector was confirmed by colony PCR on 24 randomly selected colonies using MP57 (5′-TTA TGC TTC CGG CTC GTA TG-3′) and GIII (5′-CCA CAG ACA GCC CTC ATA G-3′) primers.

### 2.2. Selection of KPC-3-Specific Nbs by Phage Display

Phage display is a widely used technology for selecting Nbs from immune libraries [22]. In this study, the constructed VHH library was panned against the KPC-3 target enzyme using M13KO7 helper phage as previously described [23]. Briefly, 10^12^ pfu/mL of helper phage was used to infect 1 mL of the VHH library. Following incubation, cultures were harvested by centrifugation, and the pellets were resuspended in 300 mL of 2xYT medium supplemented with 100 µg/mL ampicillin and 70 µg/mL kanamycin, then incubated overnight at 37 °C. The following day, phage particles were precipitated using 20% (*w*/*v*) polyethylene glycol PEG/NaCl solution and quantified using a spectrophotometer (Multiskan EX, Thermo Electron Corporation, Waltham, MA, USA) at 260 nm. Three consecutive rounds of biopanning were performed on 96-well microtiter plates (Nunc Maxisorp Plates, HTDS, Tunis, Tunisia) pre-coated with 10 µg of KPC-3 to enrich for high-affinity binders. After overnight incubation, plates were washed with phosphate-buffered saline (PBS) containing 0.05% Tween-20 (Sigma-Aldrich^®^) and blocked with 5% skimmed milk. The phage library (10^11^ pfu/mL) was added to both coated and non-coated wells. Following each round, bound phages were eluted using 100 mM triethylamine (pH 10.0, Cat. No. T0886, Sigma-Aldrich, St. Louis, MO, USA) and immediately neutralized with 1 M Tris-HCl (pH 8.0, Sigma-Aldrich^®^). The eluted phages were then used to infect log-phase *E. coli* TG1 cells, which were subsequently superinfected with M13KO7 helper phage (10^9^ pfu/mL) for the next round.

### 2.3. Identification of Positive Clones by ELISA

Following the second and third rounds of panning, 48 individual clones were randomly selected and cultured in 24-well plates containing 1 mL of 2xYT medium supplemented with 100 µg/mL ampicillin. Expression of Nb was induced with 1 mM IPTG, and periplasmic extracts were obtained from each clone using osmotic shock with TES buffer (0.5 M sucrose, 0.2 M Tris-HCl (pH 8.0), 0.5 mM EDTA) followed by TES/4. The binding capacity of the expressed Nbs toward recombinant KPC-3 was then evaluated by solid-phase ELISA. Briefly, Nunc Maxisorp plates were coated overnight at 4 °C with KPC-3 (1 µg/mL). The next day, wells were washed 5–6 times with PBS containing 0.1% Tween-20, and blocked for 2 h with 5% (*w*/*v*) skimmed milk to prevent non-specific interactions. Periplasmic extracts containing Nbs were added and incubated for 1 h at 37 °C. Bound Nbs were detected using a rabbit anti-His tag IgG antibody (1:5000, Cat. No. ab9108, Abcam, Waltham, MA, USA), followed by an HRP-conjugated goat anti-rabbit IgG antibody (1:5000, Cat. No. ab6721, Abcam, USA). The enzymatic reaction was developed with 3,3′,5,5′-tetramethylbenzidine (TMB; Abcam) and stopped by the addition of 2 N H_2_SO_4_. Absorbance was measured at 450 nm using a microplate reader.

### 2.4. VHH Sequencing

Recombinant plasmids were extracted from positive clones and sequenced using an ABI Prism 3100 Genetic Analyzer (Applied Biosystems, Waltham, MA, USA). Sequencing reactions were performed with the ABI PRISM™ BigDye Terminator v3.1 Cycle Sequencing Kit (Applied Biosystems, catalog number 4337454, USA) following the manufacturer’s protocol. MP57 and GIII primers, designed to anneal around the cloning site and flank the insert sequence, were employed for amplification. PCR was carried out for 25 cycles, consisting of an initial denaturation at 94 °C for 10 min, followed by denaturation at 96 °C for 10 s, annealing at 55 °C for 5 s, and extension at 60 °C for 4 min. The resulting chromatograms were analyzed with Sequencing Analysis Software v5.1, and sequences were aligned using the Basic Local Alignment Search Tool (BLAST+ 2.15.0, NCBI) for accurate annotation. Numbering was standardized according to the IMGT system, allowing the precise identification of FRs and CDRs [24].

### 2.5. Production and Purification of KPC-3-Specific Nb

The recombinant plasmid with the correct VHH sequence was extracted from an overnight pre-culture using the Zyppy™ Plasmid Miniprep Kit (Cat. No.: D4036, ZYMO RESEARCH, Irvine, CA, USA), and subsequently transformed via thermal shock into freshly prepared *E. coli* WK6 competent cells. The pMECS vector harbors an N-terminal PelB leader sequence, enabling the inducible secretion of soluble Nbs into the periplasm of the WK6 strain, as well as a C-terminal HA-His_6_ tag for downstream purification. For Nb expression, a freshly transformed colony was inoculated into 1 L of Terrific Broth (TB) medium and distributed into 330 mL culture flasks. Each culture was supplemented with 100 μg/mL ampicillin, 2 mM MgCl_2_, and 0.1% glucose. Bacterial growth was monitored by measuring the optical density at 600 nm (OD_600_) until it reached 0.6–0.9, after which Nb expression was induced with 1 mM IPTG (IPTG BioChemica, Cat. No.: 367-93-1, AppliChem GmbH, Darmstadt, Germany), and cultures were incubated overnight at 28 °C. Periplasmic proteins were extracted by osmotic shock, using TES buffer for 1 h, followed by TES/4 for 2 h. After centrifugation (8000 RPM, 30 min), the protein-containing supernatant was recovered and dialyzed overnight (VISKING^®^ dialysis tubing, SERVA, Electrophoresis GmbH, Heidelberg, Germany) against buffer (20 mM sodium phosphate, 500 mM NaCl, 40 mM imidazole, pH 7.4). The dialyzed fraction was then loaded onto a HisTrap™ HP affinity chromatography column (Cytiva, Marlborough, MA, USA), and recombinant Nb was eluted using an imidazole gradient (0–100%) in buffer (20 mM sodium phosphate, 500 mM NaCl, 500 mM imidazole, pH 7.4) [25]. Eluted fractions were further dialyzed into phosphate buffer (50 mM sodium phosphate, pH 7.2) to remove residual imidazole, concentrated using Amicon Ultra centrifugal filter units with a 3 kDa molecular weight cutoff (Ultracel-3K Membrane, REF: UFC500324, Merck Millipore, Carrigtwohill, Ireland), and quantified with a Bradford assay kit (Bio-Rad, Cat. No.: 500-0006, München, Germany) [26]. The purity of the Nb fraction was verified by SDS-PAGE on a 16% gel.

### 2.6. ELISA-Based Nb Binding Assay on KPC-3 Recombinant Enzyme

An ELISA was performed to evaluate the binding ability and specificity of the selected Nb to the KPC-3 recombinant enzyme. For this purpose, a 96-well Maxisorp plate was pre-coated with 1 μg/mL KPC-3. NDM-1 (New Delhi metallo-β-lactamase), BotG50 (Buthus occitanus tunetanus scorpion venom), and BSA (Bovine Serum Albumin) (1 μg/mL) were also pre-coated to confirm the binding specificity. After overnight incubation, the plate was washed 5-6 times with PBS containing 0.1% Tween-20 and blocked with 5% skimmed milk. 100 μL of Nb (1 μg/mL) was added to the antigen-coated and non-coated wells used as a control. The antigen-Nbs complexes were detected with a rabbit anti-His IgG antibody diluted at 1:5000, followed by an HRP-goat anti-rabbit IgG antibody (1:5000). After the washing step, 100 μL of TMB solution was added, and incubated for 20 min. The enzyme reaction was stopped by 50 μL of H_2_SO_4_, and absorbance values were read at 450 nm.

Following the validation of the binding ability of the selected Nb towards the KPC-3 enzyme (1 µg/mL), an ELISA dose–response was conducted to determine the half-maximal effective concentration (EC_50_) value using serial dilutions of the Nb ranging from 1 ng/mL to 1 µg/mL This assay helps to determine the binding efficiency of the selected Nb to the KPC-3 enzyme. The EC_50_ was calculated using nonlinear regression analysis with GraphPad Prism software (version 9.0.0, GraphPad Software, San Diego, CA, USA).

### 2.7. ELISA-Based Nb Binding Assay on Clinical Isolate Lysates

The binding of Nb25 to lysates from clinical isolates expressing different β-lactamases was evaluated by ELISA. Pathogenic strains, including *E. coli* co-producing KPC-3 and other β-lactamases, *K. pneumoniae* producing KPC-2, and *E. coli* producing NDM-1, were cultured in 500 mL TB medium supplemented with 0.25 µg/mL meropenem until an OD_600_ of ~0.9 was reached (~2 h at 37 °C, 220 RPM). Cells were harvested by centrifugation at 4000 RPM for 30 min at 4 °C. The resulting pellets were washed twice with 25 mM sodium phosphate buffer (NaPO_4_, pH 7.0) and centrifuged for 10 min at 8000 RPM. Periplasmic proteins were extracted using TES buffer (50 mM Tris-HCl, pH 8.0, 0.5 mM EDTA, and 20% [*w*/*v*] sucrose). The cell suspensions were incubated under gentle shaking at 4 °C for 30 min. Subsequently, MgSO_4_ solution was added to a final concentration of 5 mM, and incubation continued under the same conditions. The mixtures were centrifuged at 15,000 RPM for 30 min at 4 °C, and the resulting supernatants containing periplasmic proteins were collected. Protein concentrations were determined using the Bradford assay.

Subsequently, periplasmic extracts were diluted in carbonate–bicarbonate coating buffer (pH 9.6) at increasing concentrations, and used to coat 96-well MaxiSorp™ plates (Thermo Fisher Scientific, Waltham, MA, USA) overnight at 4 °C. Extracts from the *E. coli* strain producing NDM-1, and non-coated wells served as negative controls, while purified KPC-3 protein was included as a positive control (not shown in graphs). A fixed concentration of Nb25 (2 µg/mL) was added to each well. Plates were washed five times with PBS containing 0.1% (*v*/*v*) Tween-20, and subsequently blocked with 3% (*w*/*v*) BSA (Catalog number A9647, Sigma-Aldrich^®^, St. Louis, MO, USA) for 1.5 h at 37 °C. For detection, a mouse monoclonal anti-HA antibody (diluted at 1:4000) was applied, followed by an HRP-conjugated anti-mouse secondary antibody (diluted at 1:5000). All antibodies were diluted in PBS containing 0.1% (*v*/*v*) Tween-20 supplemented with 0.1% BSA. After final washes, signal development was carried out using 100 μL TMB substrate (catalog number T8665, Sigma-Aldrich^®^), and the enzymatic reaction was stopped after 30 min by adding 2 N H_2_SO_4_. Absorbance was measured at 450 nm using a Tecan Infinite^®^ 200 PRO M Plex Microplate Reader (Männedorf, Switzerland).

### 2.8. Western Blot Validation of Nb25 Specificity

The binding of Nb25 to recombinant KPC-3, and to lysates from clinical isolates expressing different β-lactamases [*E. coli* (NDM-1), *K. pneumoniae* (IMP-1, SHV-12, OXA-1, CTX-M-15, OXA-48, KPC-2, and *E. coli* (KPC-3 co-expressed with other β-lactamases)] was evaluated by Western blot. First, bacterial colonies were cultured in LB medium until an OD_600_ of 0.9 was reached, and harvested by centrifugation (4000 RPM, 30 min, 4 °C). Pellets were washed twice with 25 mM sodium phosphate buffer (NaPO_4_, pH 7.0), and resuspended in 30 mM Tris-HCl buffer (pH 8.0). Cells were treated with lysozyme (0.4 mg/mL) and EDTA (5 mM) for 50 min on ice, followed by the addition of magnesium sulfate solution (MgSO_4_, 5 mM). After centrifugation (15,000 RPM, 45 min, 4 °C), periplasmic proteins were recovered from the supernatant, while the pellets were resuspended in 25 mM NaPO_4_ and subjected to five cycles of sonication to release cytoplasmic proteins. Supernatants were collected after centrifugation (15,000 RPM, 45 min), and protein concentrations were determined using the Bradford assay. Subsequently, purified KPC-3 (125, 250, and 500 ng), Nb25 (500 ng, control), periplasmic (20 µg), and cytoplasmic (25 µg) protein extracts were separated on 12% SDS-PAGE gels and transferred onto PVDF membranes (Bio-Rad, Cat. No. 162-0177). Transfer efficiency was verified with Ponceau S staining. Membranes were blocked for 1 h with 3% (*w*/*v*) BSA (Sigma-Aldrich, Cat. No. A9647), and washed three times with TBS-T (20 mM Tris, pH 7.5; 500 mM NaCl; 0.1% Tween-20). For KPC-3 detection, membranes were incubated overnight at 4 °C with Nb25 (2 µg/mL), followed by 2 h incubation at room temperature with a monoclonal anti-HA antibody (Sigma-Aldrich, Cat. No. H9658, 1:20,000), and 1 h with HRP-conjugated anti-mouse IgG secondary antibody (Thermo Fisher Scientific, 1:7000). Chemiluminescence was developed using WesternBright™ ECL (Advansta, Cat. No. K-12045-D50, Advansta Inc., Menlo Park, CA, USA) and visualized with the ChemiDoc™ MP Imaging System (Bio-Rad). A prestained protein ladder (SHARPMASS^TM^ VII, 6.5–270 kDa, Cat. No. EPS026500, Euroclone, Milan, Italy) served as a reference.

### 2.9. Inhibition Kinetic Assays

The inhibitory potency of Nb25 against recombinant KPC-3 was evaluated by monitoring meropenem hydrolysis using a Perkin-Elmer Lambda 25 spectrophotometer. Nb25 at varying concentrations (0–100 µM) was mixed with 100 µM meropenem in 25 mM PB buffer (pH 7.2), and the reaction was initiated by adding 60 µM KPC-3. To assess time-dependent inhibition, mixtures containing fixed concentrations of KPC-3 (60 µM) and Nb25 (100 µM) were preincubated at room temperature for 0, 10, 20, 30, 45, 60, 75, 90, 120, 180, or 240 min before measuring hydrolysis rates.

### 2.10. Structural Bioinformatics Investigations

The three-dimensional (3D) structures of the anti-KPC-3 nanobody Nb25 and the KPC-3 β-lactamase (receptor) were employed for molecular docking studies. The Nb structure was first generated through comparative modeling using SWISS-MODEL [27], based on its amino acid sequence, prior to refinement and docking. Before docking, all structures were prepared by removing water molecules, heteroatoms, and bound ligands to retain only the protein chains. Structural refinement was performed using FoldX to optimize side-chain conformations and minimize steric clashes [28]. Model validation was carried out using ERRAT to assess non-bonded atomic interactions [29], Verify3D to evaluate residue environment compatibility [30], and Ramachandran plot analysis to ensure correct dihedral angle distributions. Additional structural stabilization was performed using the MMTSB Tool Set [31]. Docking was performed using HADDOCK 2.4, where nanobody CDR residues were defined as active residues and neighboring residues within 6 Å were set as passive residues. The docking workflow followed the standard three-step protocol: rigid-body docking (it0), semi-flexible refinement (it1), and explicit water refinement. The best-ranked cluster was selected based on HADDOCK score, van der Waals energy, electrostatic energy, and RMSD values.

Binding affinity was predicted using PRODIGY (web server) [32], which estimated the binding free energy (ΔG) and dissociation constant (*Kd*) based on interfacial contacts and non-covalent interactions at 25 °C. Interaction profiles, including hydrogen bonding, hydrophobic contacts, and nanobody–receptor interface characterization, were visualized using PyMOL 2.5.4 (Schrödinger, LLC).

## 3. Results

### 3.1. VHH Library Construction

**Figure 1:**
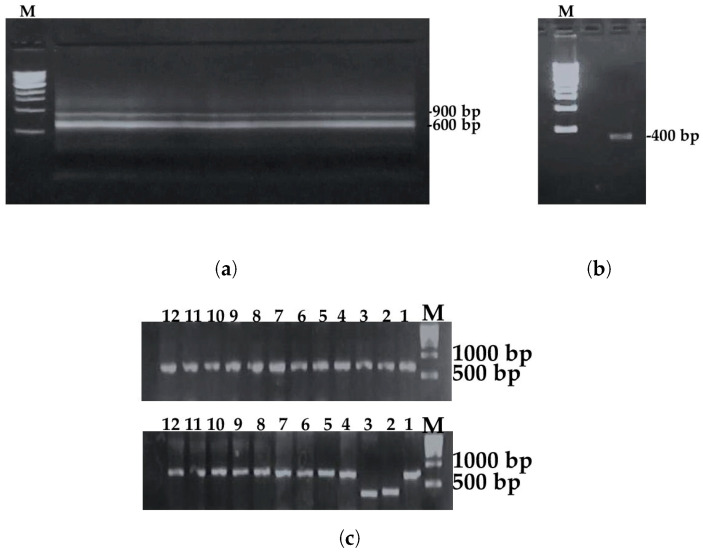
Construction of the VHH library. (**a**,**b**) Two-step PCR amplification of VHH sequences. The first PCR (CALL001/CALL002) amplified both conventional antibody VH–CH1–hinge–CH2 fragments (900 bp) and camelid heavy-chain antibody (HCAb) VHH–hinge–CH2 fragments (600 bp) (**a**). The nested PCR (A6E/PMCF) selectively amplified the VHH domains, yielding a 400 bp band (**b**). (**c**) Colony PCR of 24 randomly selected TG1 transformants to estimate library quality and correct VHH insertion (~700 bp) in the pMECS phage display vector. M: DNA marker (SHARPMASS^TM^ 1Kb, Cat. No. EMR815100). Western blot original images can be found in Supplementary Materials.

The DNA encoding the VHH sequences was successfully amplified through a two-step PCR process (Figure 1a,b). (The resulting VHH DNA was cloned into the pMECS expression vector and transformed into the suppressor TG1 strain. Serial dilutions of the transformed bacteria were used to estimate library size, and 24 randomly selected colonies were analyzed by PCR. Our immune VHH library contained over 10^6^ individual transformants, with more than 90% of clones harboring a phage display vector containing an insert with optimal VHH size (~700 bp) (Figure 1c) [33].

### 3.2. Panning Phage Display

**Figure 2:**
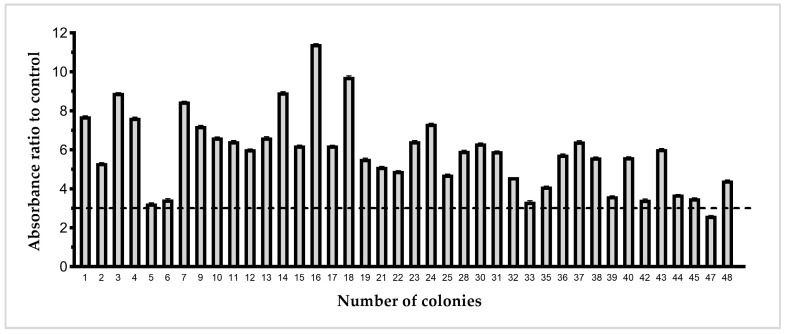
Selection of KPC-3-specific Nb clones by PE-ELISA. Periplasmic extracts from 48 randomly selected clones were tested against KPC-3-coated wells. Non-coated wells were used as negative controls. Clones showing an absorbance ratio > 3 compared to the control were scored as positive.

Three consecutive rounds of phage display biopanning were performed to isolate KPC-3-specific Nbs. After panning, 48 clones were randomly selected and cultured in 2×TY medium containing ampicillin. Protein expression was induced with 1 mM isopropyl-β-D-thiogalactopyranoside (IPTG) at an OD_600_ of 0.6–0.9, and cells were incubated overnight at 28 °C. Periplasmic proteins were extracted by osmotic shock and analyzed by ELISA. Non-antigen-coated wells served as negative controls, and clones exhibiting an absorbance ratio greater than three relative to the control were considered positive (Figure 2).

### 3.3. VHH Sequencing Analysis

Sequence analysis of positive clones revealed a single dominant sequence, designated Nb25. The recovery of a single dominant clone reflects the limited diversity of the library (~10^6^ CFU/mL), which reduces the likelihood of multiple binders, and the strong enrichment of Nb25, likely due to its high intrinsic affinity under the stringent panning conditions [34].

**Figure 3:**
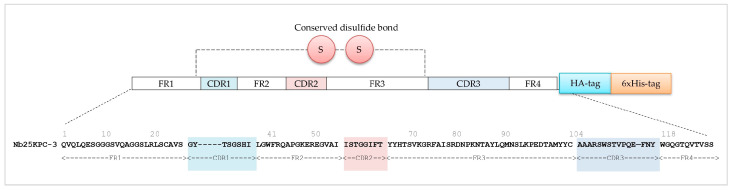
Nb25 sequence analysis. CDRs are highlighted: CDR1 in pink, CDR2 in orange, and CDR3 in blue. These regions are essential for antigen binding and define the specificity of the Nb. Tags used for detection and purification are also indicated. The two conserved cysteine residues are schematically represented.

The sequence was annotated into framework regions (FR1–FR4) and complementarity-determining regions (CDR1–CDR3) according to the International ImMunoGeneTics (IMGT) information system^®^ [24] (Figure 3). Nb25, comprising 122 amino acids (excluding tag sequences), was compared with other Nb sequences using NCBI Protein BLAST and showed high homology with VHH sequences derived from *Camelus*. Both CDR1 and CDR2 contain 8 amino acids, consistent with typical Nb lengths, while CDR3 is notably long at 15 residues, often critical for antigen recognition. The sequence retains all characteristic VHH residues, including Phe42 (F), Glu49 (E), Arg50 (R), and Gly52 (G) in FR2, as well as the conserved Trp118 in FR4. Nb25 also contains two conventional cysteines (Cys23/Cys104) in FR1 and FR3, supporting proper structural folding and stability.

### 3.4. ELISA-Based Nb Binding Assay on Recombinant KPC-3

The single VHH-encoded sequence was transformed into *E. coli* WK6 cells for periplasmic expression. The soluble Nb was purified using a HisTrap™ HP 5 mL column and eluted with a 60% imidazole gradient (~300 mM). The eluted fraction was dialyzed against 50 mM sodium phosphate buffer (pH 7.2) and concentrated tenfold. Nb25 production yield was estimated at 2.7 mg/L of TB medium. Its molecular weight (MW), theoretical isoelectric point (pI), and extinction coefficient (ε) were calculated using the ExPASy ProtParam Tool (Table 1).

**Figure 4:**
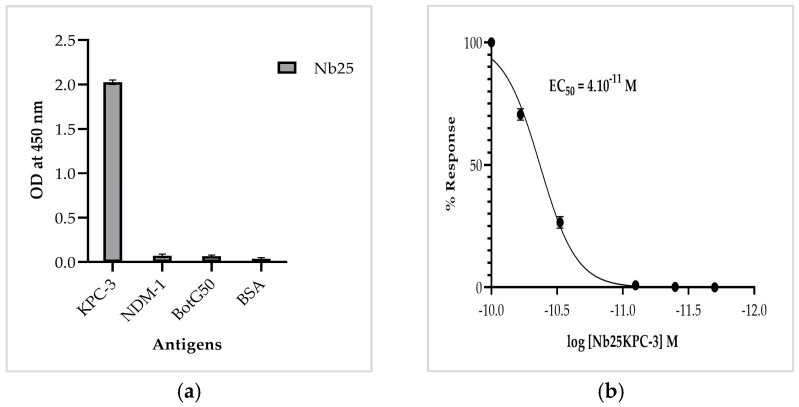
Binding specificity and affinity of Nb25 toward KPC-3 recombinant enzyme. (**a**) Specificity of Nb25 assessed by ELISA against KPC-3, with NDM-1, BotG50, and BSA recombinant proteins as controls. (**b**) Dose–response curve of Nb25 binding to KPC-3, showing high-affinity interaction (*EC*_50_ = 4 × 10^−11^ M). All values represent the mean of triplicate measurements.

ELISA was subsequently performed to evaluate the specificity and the binding efficiency of the purified Nb against recombinant KPC-3. Nb25 (5 μg/mL) exhibited strong and specific binding to the KPC-3 enzyme (1 μg/mL, OD_450_ = 2) with negligible reactivity toward NDM-1, BotG50, or BSA (Figure 4a). To further assess binding efficiency, an ELISA was performed with serial dilutions of Nb25. The resulting dose–response curve revealed strong binding, with an EC_50_ of 4 × 10^−11^ M, which reflects binding potency rather than a true kinetic affinity constant. (Figure 4b). All ELISA experiments were performed three times, yielding consistent results.

### 3.5. Results of ELISA-Based Nb Binding Assay on Clinical Isolate Lysates

**Figure 5:**
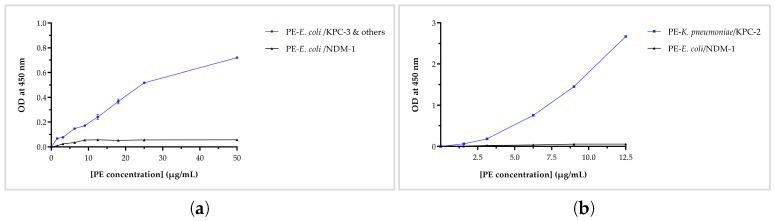
ELISA-based analysis of Nb25 binding to periplasmic extracts from clinical isolates. (**a**) Binding specificity of Nb25 toward KPC-3 using periplasmic extracts from *E. coli* expressing KPC-3, with *E. coli* expressing NDM-1 as a negative control. (**b**) Binding of Nb25 to KPC-2 using periplasmic extracts from *K. pneumoniae* expressing KPC-2, with *E. coli* producing NDM-1 as a cross-reactivity control. Error bars represent standard deviation (SD) from triplicate measurements; due to minimal variability, some may be visually negligible.

Due to the strong binding affinity of Nb25 for the recombinant enzyme, we next aimed to assess its ability to detect KPC-3 in clinical isolates. The binding specificity of Nb25 was assessed using ELISA with serial dilutions of periplasmic extracts from clinical isolates. Nb25 specifically recognized KPC-3 in *E. coli*, reaching an OD_450_ of 0.7 at 50 µg/mL, and showed no detectable cross-reactivity toward NDM-1 (Figure 5a). We then extended the analysis to KPC-2 in *K. pneumoniae*, where Nb25 generated a stronger signal exceeding 2 OD units at a lower concentration of 12.5 µg/mL (Figure 5b). All ELISA experiments were performed three times, yielding consistent results.

### 3.6. Western Blot Analysis of Nb25 Binding Specificity

**Figure 6:**
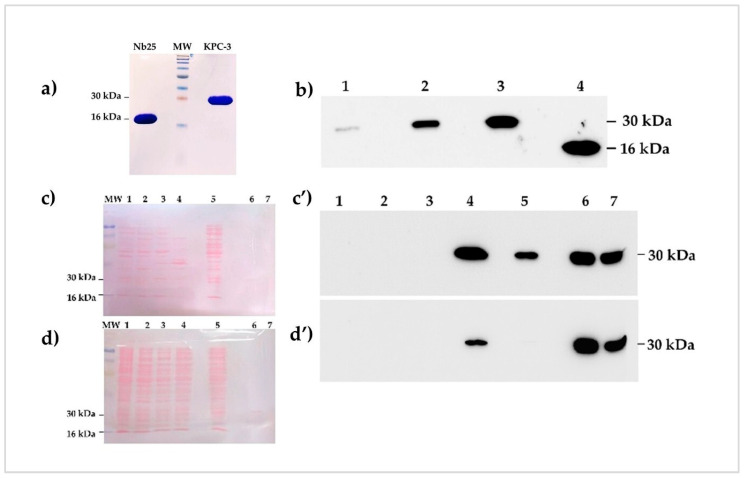
Western blot analysis of Nb25 specificity for recombinant KPC-3 and clinical isolate lysates. (**a**) SDS-PAGE (16%) of purified proteins showing distinct bands for Nb25 (~16 kDa) and KPC-3 (~30 kDa), confirming purity. (**b**) Western blot demonstrated concentration-dependent binding of Nb25 to denatured KPC-3. Lanes 1–3: 125, 250, 500 ng KPC-3; lane 4: 500 ng Nb25 alone (control). (**c**) Ponceau S staining of periplasmic extracts (20 µg/lane) from clinical bacterial strains. Lane 1: *E. coli* (NDM-1); lane 2: *K. pneumoniae* (IMP-1, SHV-12); lane 3: *K. pneumoniae* (OXA-1, CTX-M-15, OXA-48); lane 4: *K. pneumoniae* (KPC-2); lane 5: *E. coli* (KPC-3 + others); lane 6: recombinant *E. coli* BL21 expressing KPC-3; lane 7: purified KPC-3. (**c’**) Corresponding Western blot probed with Nb25 (2 µg/mL) and anti-HA antibody (1:20,000), followed by HRP-conjugated secondary antibody (1:7000). (**d**) Ponceau S staining of cytoplasmic extracts (25 µg/lane) from the same strains. (**d’**) Corresponding Western blot showing Nb25 binding. Western blot original images can be found in Supplementary Materials.

The purified Nb25 was verified by SDS-PAGE, which showed a single band at ~16 kDa (Figure 6a). Western blot analysis further confirmed its specific binding to the HA-tag antibody (Figure 6b, lane 4). The specificity of Nb25 was further assessed by Western blotting. Nb25 (2 µg/mL) detected purified KPC-3 in a concentration-dependent manner (125–500 ng; Figure 6b, lanes 1–3). Ponceau S staining was used to verify efficient protein transfer to the membrane (Figure 6c,d). Remarkably, Nb25 also recognized KPC-3 in periplasmic extracts from a pathogenic *E. coli* strain (Figure 6c’, lane 5). Densitometric analysis using Image Lab™ v6.0.1 (Bio-Rad) estimated that KPC-3 comprised ~14.4% of the total periplasmic protein, corresponding to ~2.88 µg. A faint band observed in lane 5 of Figure 6d’ likely reflects periplasmic export of most KPC-3, consistent with Figure 6c’, lane 5. Cross-reactivity with KPC-2 was observed (Figure 6c’,d’, lane 4), likely due to the high sequence similarity between KPC-2 and KPC-3 (differing only by H274Y). Importantly, no cross-reactivity of Nb25 was observed with bacterial cells expressing IMP-1, SHV-12, OXA-1, CTX-M-15, or OXA-48, which lack KPC-2 and KPC-3 (Figure 6c’,d’, lanes 1–3). All WB experiments were performed three times.

### 3.7. Kinetic Analysis

Kinetic assays measuring meropenem hydrolysis were performed to evaluate the inhibitory potential of Nb25. Increasing concentrations of Nb25 (up to 100 μM) did not significantly affect the substrate hydrolysis rate, and pre-incubation with KPC-3 did not improve inhibitory activity. These results indicate that Nb25 does not function as a KPC-3 inhibitor. Nevertheless, its strong binding affinity to the enzyme underscores its potential for alternative diagnostic applications and supports future investigations, including mutagenesis studies aimed at enhancing inhibitory capability.

### 3.8. Predicted Structural Analysis of the Nb25/KPC-3 Complex

Docking analysis of the Nb25–KPC-3 complex revealed a well-defined and highly convergent interaction interface. The top HADDOCK cluster displayed a favorable overall score (−110.5 ± 5.2) with strong van der Waals and electrostatic contributions, and a low internal RMSD (1.8 Å), indicating a stable and reproducible binding mode. PRODIGY predicted a high-affinity interaction (ΔG ≈ −11.7 kcal·mol^−1^; *Kd* ≈ 2.6 nM).

**Figure 7:**
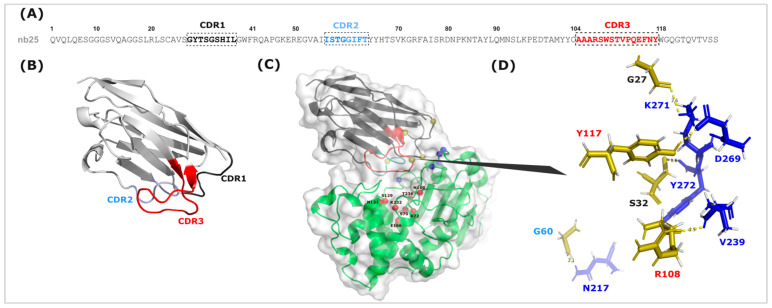
Structural and docking analysis of Nb25 with KPC-3. (**A**) Amino acid sequence of Nb25, with CDR1, CDR2, and CDR3 highlighted. (**B**) Three-dimensional model of Nb25 showing the overall β-sheet framework and the three CDR loops (CDR1 in black, CDR2 in blue, and CDR3 in red). (**C**) Docking model of the Nb25–KPC-3 complex. Nb25 is shown in ribbon representation interacting with the surface of KPC-3. Red spheres indicate the catalytic residues of KPC-3, blue spheres highlight Nb25 residues involved in binding, and gold spheres represent the KPC-3 residues contacting Nb25. (**D**) Close-up view of the interaction interface, illustrating the main contacting residues between Nb25 (yellow) and KPC-3 (blue), with hydrogen bonds indicated by dashed lines.

The binding interface is formed predominantly by the three CDR loops (Figure 7). CDR1 contributes anchoring contacts with residues K271 and Y272 of KPC-3, whereas CDR2 makes a minor stabilizing contribution through a polar interaction between G60 and N217. CDR3 is the main determinant of recognition: residues Y117 and R108 form key interactions with D269 and V239, respectively, defining a lateral epitope on the enzyme. Importantly, the identified epitope lies outside the catalytic region of KPC-3. No CDR residues interact with the catalytic triad (S70, K73, S130) or the essential deacylation residue E166. The orientation of Nb25 does not obstruct access to the active-site groove, indicating that its binding does not interfere with the geometry of the catalytic machinery.

## 4. Discussion

Among the various fields where Nbs have shown remarkable potential, infectious diseases remain one of the most significant and rapidly advancing areas of application [35,36,37]. Owing to their strong ability to target enzyme active sites, Nb-based technologies have emerged as effective tools for both detecting and inhibiting β-lactamases [16,17]. Notably, our team has previously generated Nbs against NDM-1, which proved to be highly potent inhibitors [16]. Building on this experience, we aimed to extend our approach to KPC-type carbapenemases, focusing on the development of high-affinity binders for diagnostic detection. In this study, we successfully obtained a VHH library of 10^6^ CFU/mL with over 90% of the clones harboring a phage display vector having an insert with the best size of a VHH gene (approximately 700 bp). It has been proven that a modest-sized VHH library of 10^6^ individual transformants easily yields high-affinity binders after one or two rounds of panning [38]. Consistent with this, three rounds of phage display biopanning, the most common approach for Nb selection [39,40], followed by ELISA screening, allowed us to identify strong binders against KPC-3. Remarkably, sequencing revealed that all positive clones corresponded to a single sequence, designated as Nb25, which exhibited hallmark VHH residues. The structural analysis of CDRs indicated typical Nb features, including short CDR1/CDR2 (8 aa each) and a relatively long CDR3 (15 aa), a characteristic frequently associated with enhanced antigen specificity and affinity [41]. ELISA assays on recombinant enzymes revealed that Nb25 displayed strong and specific binding to KPC-3, without cross-reactivity toward NDM-1, BotG50, or BSA. A dose–response ELISA further confirmed its high binding for recombinant KPC-3, with an EC_50_ of 4 × 10^−11^ M. Nevertheless, precise kinetic parameters will require independent validation using biophysical methods such as SPR. Encouraged by these results, we first evaluated Nb25 against KPC-3 in *E. coli* clinical isolates. Given the potential of Nbs to recognize variants within the same enzyme family [17], we subsequently tested Nb25 against KPC-2 in *K. pneumoniae*. Interestingly, the ELISA signal was higher for KPC-2 in *K. pneumoniae* than for KPC-3 in *E. coli,* with no cross-reactivity toward NDM-1. Since KPC-2 and KPC-3 differ by only a single amino acid [42], this subtle variation may affect local conformation and epitope accessibility, thereby enhancing Nb25 binding to KPC-2. Alternatively, KPC-2 might be expressed at higher levels in *K. pneumoniae* isolates, which could also account for the stronger signal. Finally, differences in the periplasmic environments of *E. coli* and *K. pneumoniae* may influence enzyme folding and presentation. Further structural and quantitative analyses will be required to clarify the basis of this enhanced recognition. This combination of intra-family recognition and inter-family selectivity is advantageous for diagnostic purposes, as cross-reactivity remains a major challenge in β-lactamase detection assays. The combination of strong binding affinity and stringent specificity makes Nb25 a promising candidate for diagnostic applications [43], with potential for integration into Nb-based electro-chemiluminescent immunosensors for sensitive detection of KPC variants [44]. Western blot analysis, being more discriminative than ELISA, demonstrated that Nb25 specifically recognized KPC-3 at different concentrations with high affinity. An important aspect of this study is the validation of Nb25 against clinical isolates. Western blotting assays confirmed its ability to detect KPC-2 and KPC-3 enzymes in periplasmic extracts of *K. pneumoniae* and *E. coli* isolates, respectively. The strong binding of Nb25 to both KPC-3 and KPC-2 is consistent with their single amino acid difference (H274Y) and reflects recognition of a conserved KPC epitope rather than lack of specificity. This result is consistent with earlier reports highlighting the robustness of Nbs in recognizing conserved β-lactamase epitopes [17]. Interestingly, the Nb did not cross-react with unrelated β-lactamases such as NDM-1, IMP-4, SHV-12, OXA-1, CTX-M-15, and OXA-48, underscoring its stringent specificity for KPC variants. However, the selected Nb was unable to inhibit KPC-3 enzymatic activity. To explore this, we performed comprehensive bioinformatics studies integrating structural modeling, molecular docking, and interaction profiling. The molecular docking analysis predicted that Nb25 interacts with a putative epitope composed of residues located next to, but not entirely inside, the catalytic cleft. This indicates that Nb25 attaches to a conserved and accessible region of the enzyme surface rather than directly interfering with its catalytic machinery. This type of peripheral binding explains why it exhibits strong affinity without affecting enzyme function. Similar cases have been described for other nanobodies, such as anti-AahII Nbs that bind their targets with high affinity but do not neutralize their biological activity [45]. In parallel, the predicted structural model of the Nb25–KPC-3 complex explains the observed high-affinity binding without enzymatic inhibition. Nb25 binds a lateral, non-catalytic surface of KPC-3, away from the active site residues S70, K73, S130, and E166. Docking shows that CDR loops do not reach or alter the catalytic pocket, leaving substrate hydrolysis unaffected. This aligns with other non-inhibitory nanobodies that target peripheral or regulatory surfaces [18,46]. The dominant CDR3 mediates epitope recognition without steric hindrance, explaining the lack of inhibition. This limitation could be addressed through rational engineering of CDR3, or the use of multivalent/bifunctional formats could introduce steric occlusion, converting Nb25 into an inhibitor. This highlights how epitope positioning dictates inhibitory potential and guides the design of next-generation KPC-3-targeting nanobodies. Finally, it is important to emphasize that this study serves as a robust biochemical and structural proof-of-concept for protein-level detection of KPC enzymes. Unlike PCR-based methods, which detect gene presence, nanobody-based approaches directly target the expressed enzyme, potentially improving correlation with resistance phenotype. Because most KPC variants differ by only a few amino acid substitutions, Nb25 is expected to retain binding to additional members of the KPC family. Its ability to function in a highly complex, protein-rich environment complements PCR by providing a direct assessment of KPC enzyme presence in clinical samples, representing a significant step toward practical diagnostic implementation.

Building on these results, our future work will focus on developing Point-of-Care platforms (e.g., lateral flow assays and electrochemical biosensors) tailored for direct detection of KPC variants in clinical samples, including urine and serum. Its cross-reactivity with KPC-2 and KPC-3 allows detection of multiple KPC variants in a single test. Nb25 therefore constitutes both a promising pan-KPC diagnostic candidate and a versatile scaffold for further therapeutic optimization, with the potential to improve surveillance and management of antimicrobial resistance.

## 5. Conclusions

This study identifies Nb25 as the first structurally resolved nanobody with high cross-reactive binding to KPC-2 and KPC-3, class A carbapenemases, showing great potential for rapid detection of KPC-producing bacteria in clinical settings. Moreover, Nb25 represents a versatile scaffold that can be further engineered, through mutagenesis or tandem-repeat formats, to enhance inhibitory activity, opening new avenues for both rapid diagnostics and therapeutic innovation.

## Figures and Tables

**Table 1:** Properties of KPC-3-specific Nb.

VHH	MW (kDa)	pI	Ext. Coefficient ε (g/L)	Yield (mg/L)
Nb25	15.38796	8.00	2.049	2.7

## Data Availability

Data supporting reported results can be provided by the NanoBioMediKa (Pasteur Institute of Tunis, https://www.pasteur.tn/, accessed on 15 January 2026) and DISCAB (University of L’Aquila, https://discab.univaq.it, accessed on 15 January 2026) teams.

## Supplementary Materials

The following supporting information can be downloaded at: https://www.mdpi.com/article/10.3390/biom16030369/s1, Western blot original images.

## Abbreviations

The following abbreviations are used in this manuscript:

KPC	Klebsiella pneumoniae carbapenemase
VHHs	Variable domains of the Heavy chain of a Heavy-chain-only antibody
Nbs	Nanobodies
