# Supplementary material for: A High-Affinity Nanobody Selectively Recognizing KPC-2/KPC-3: Biochemical and Structural Insights
Source: Biomolecules. 2026 Feb 28;16(3):369. doi: 10.3390/biom16030369 (PMC13024528; doi:10.3390/biom16030369)
Supplement: Supplementary file 1 [file biomolecules-16-00369-s001.zip › biomolecules-4093902-wb.pdf]

A High-Affinity Nanobody Selectively Binding KPC-3: Biochemical and Structural Insights

Original Gels and Western blots :

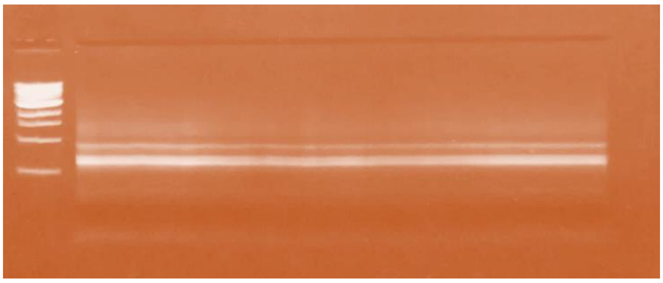

(a)

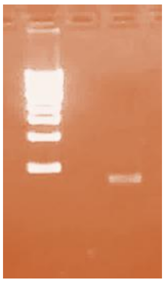

(b)

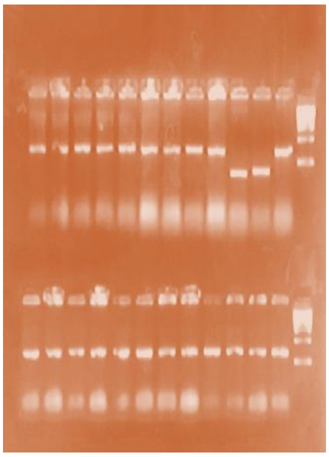

(c)

Figure 1. Construction of the VHH library. (a, b) Two-step PCR amplification of VHH sequences. The first PCR (CALL001/CALL002) amplified both conventional antibody VH–CH1–hinge–CH2 fragments (900 bp) and camelid heavy-chain antibody (HCAb) VHH–hinge–CH2 fragments (600 bp) (a). The nested PCR (A6E/PMCF) selectively amplified the VHH domains, yielding a 400 bp band (b). (c) Colony PCR of 24 randomly selected TG1 transformants to estimate library quality and correct VHH insertion (~700 bp) in the pMECS phage display vector. M: DNA marker (SHARPMASS™ 1Kb, Cat. No. EMR815100).

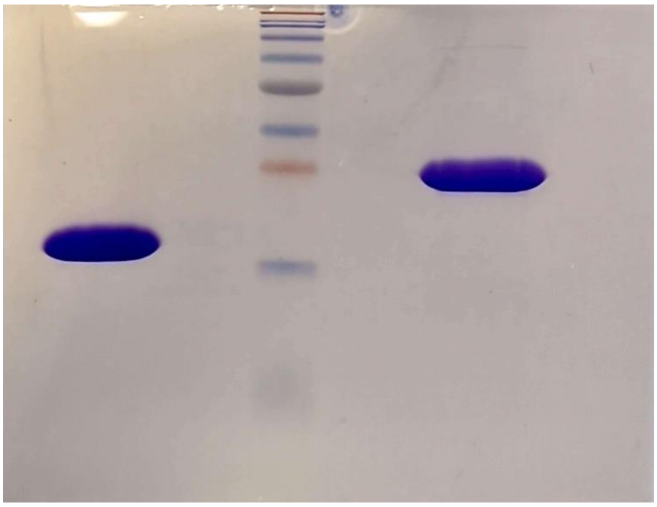

(a)

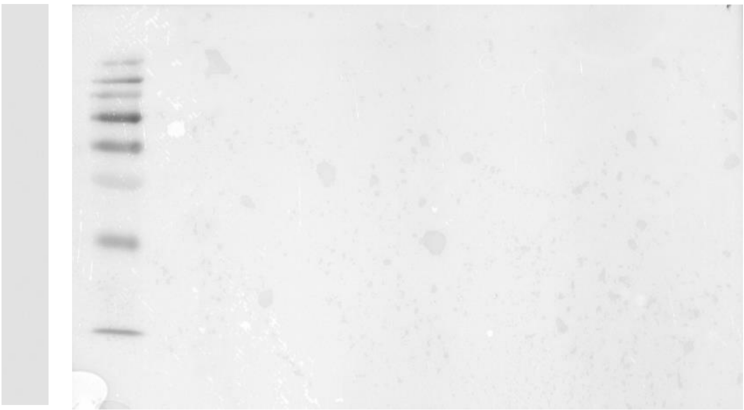

(b)

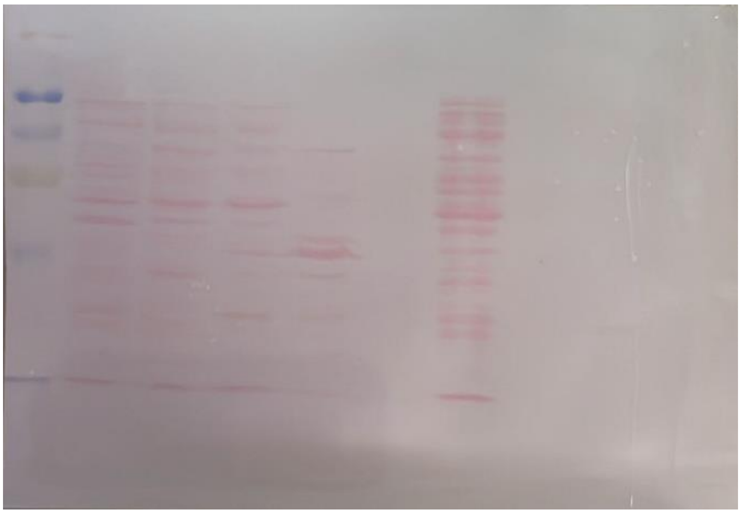

(c)

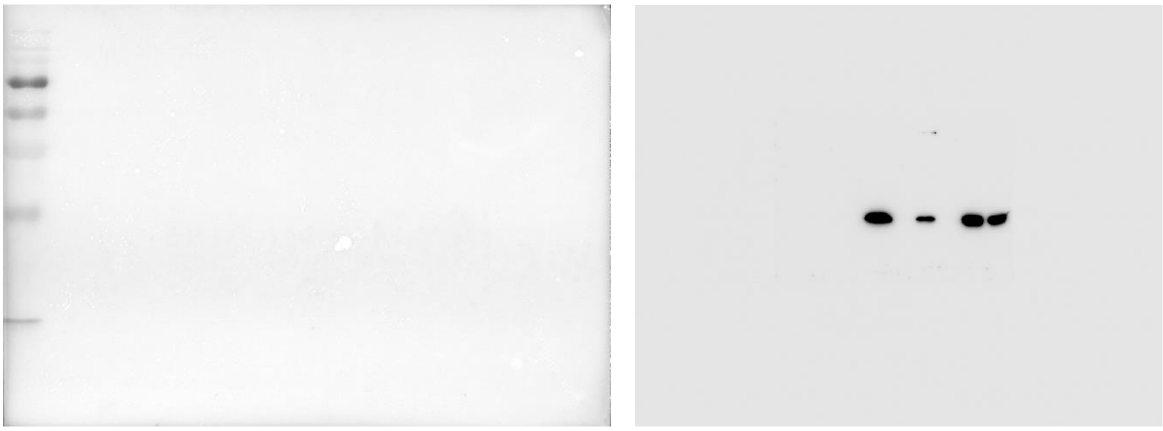

(c′)

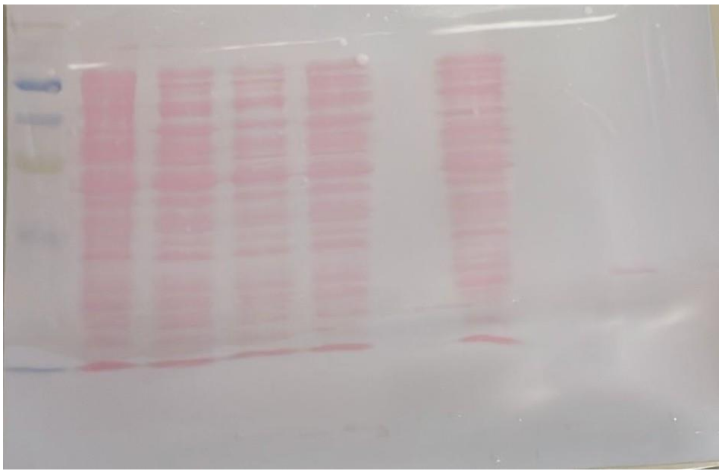

(d)

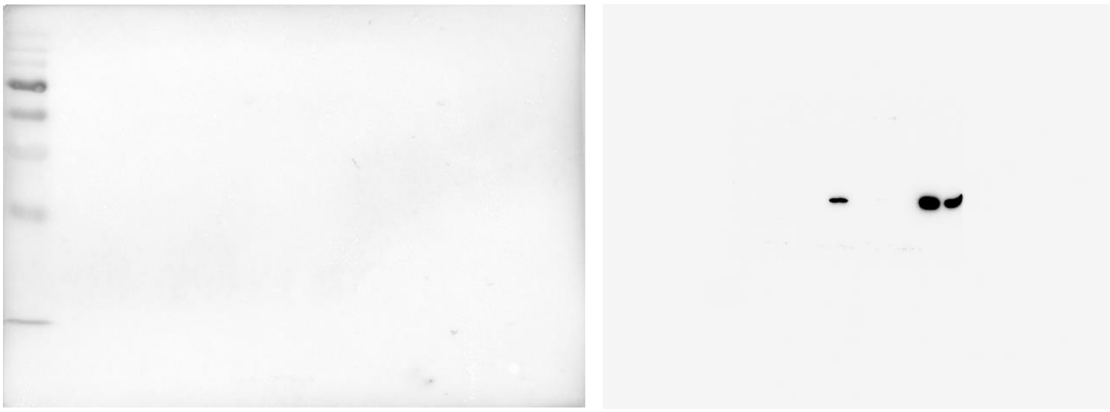

(d′)

Figure 6. Western blot analysis of Nb25 specificity for recombinant KPC-3 and clinical isolate lysates. (a) SDS-PAGE (16%) of purified proteins showing distinct bands for Nb25 (~16 kDa) and KPC-3 (~30 kDa), confirming purity. (b) Western blot demonstrated concentration-dependent binding of Nb25 to denatured KPC-3. Lanes 1–3: 125, 250, 500 ng KPC-3; lane 4: 500 ng Nb25 alone (control). (c) Ponceau S staining of periplasmic extracts (20 μg/lane) from clinical bacterial strains. Lane 1: E. coli (NDM-1); lane 2: K. pneumoniae (IMP-1, SHV-12);

lane 3: K. pneumoniae (OXA-1, CTX-M-15, OXA-48); lane 4: K. pneumoniae (KPC-2); lane 5: E. coli (KPC-3 + others); lane 6: recombinant E. coli BL21 expressing KPC-3; lane 7: purified KPC-3. (c′) Corresponding Western blot probed with Nb25 (2 µg/mL) and anti-HA antibody (1:20,000), followed by HRP-conjugated secondary antibody (1:7,000). (d) Ponceau S staining of cytoplasmic extracts (25 µg/lane) from the same strains. (d′) Corresponding Western blot showing Nb25 binding.
