# Supplementary material for: A High-Affinity Nanobody Selectively Recognizing KPC-2/KPC-3: Biochemical and Structural Insights
Source: Biomolecules. 2026 Feb 28;16(3):369. doi: 10.3390/biom16030369 (PMC13024528; doi:10.3390/biom16030369)
Supplement: Supplementary file 1 [file biomolecules-16-00369-s001.zip › Original Figures SDS PAGE_WB.pdf]

A High-Affinity Nanobody Selectively Binding KPC-3: Biochemical and Structural Insights

Figure 6a:

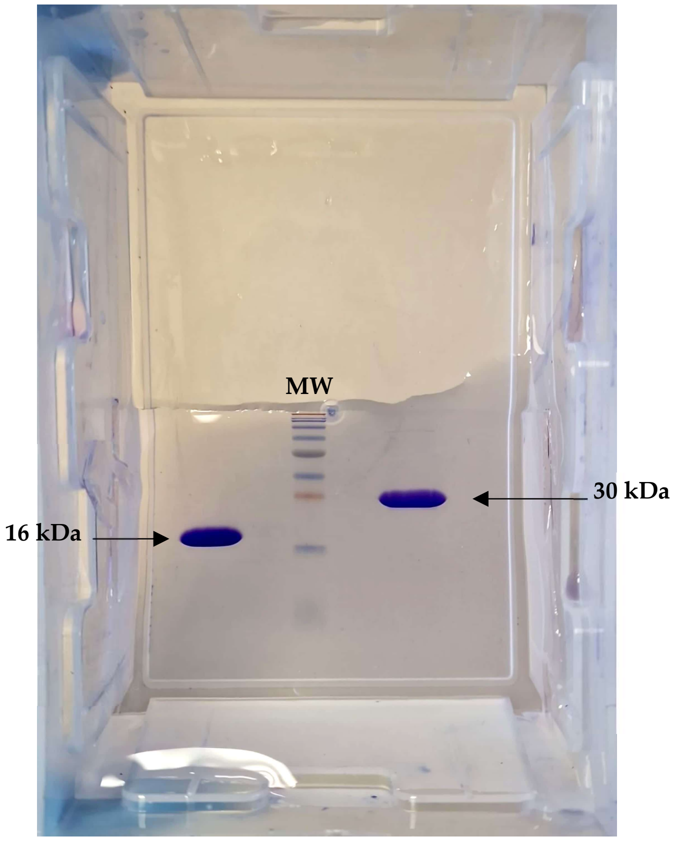

(a)

Figure 6b before the merge:

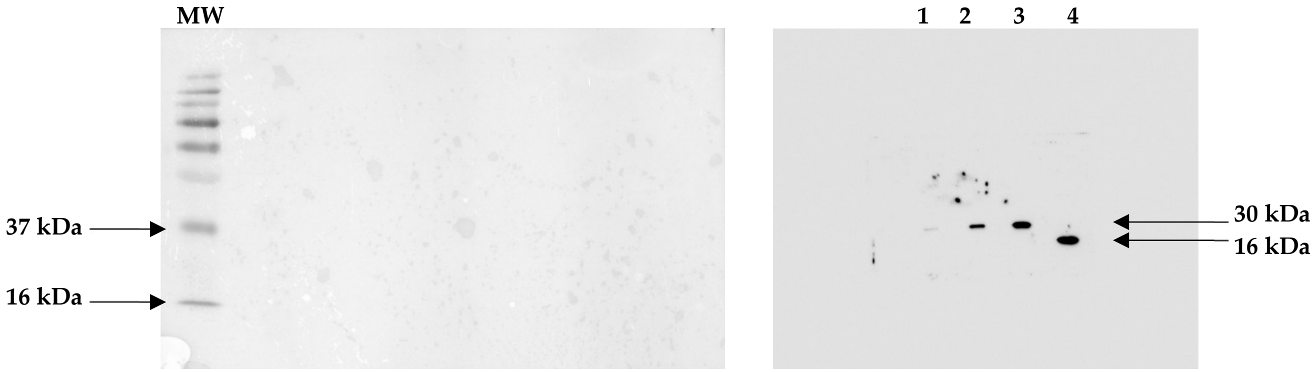

(b) before the merge

Figure 6b after the merge:

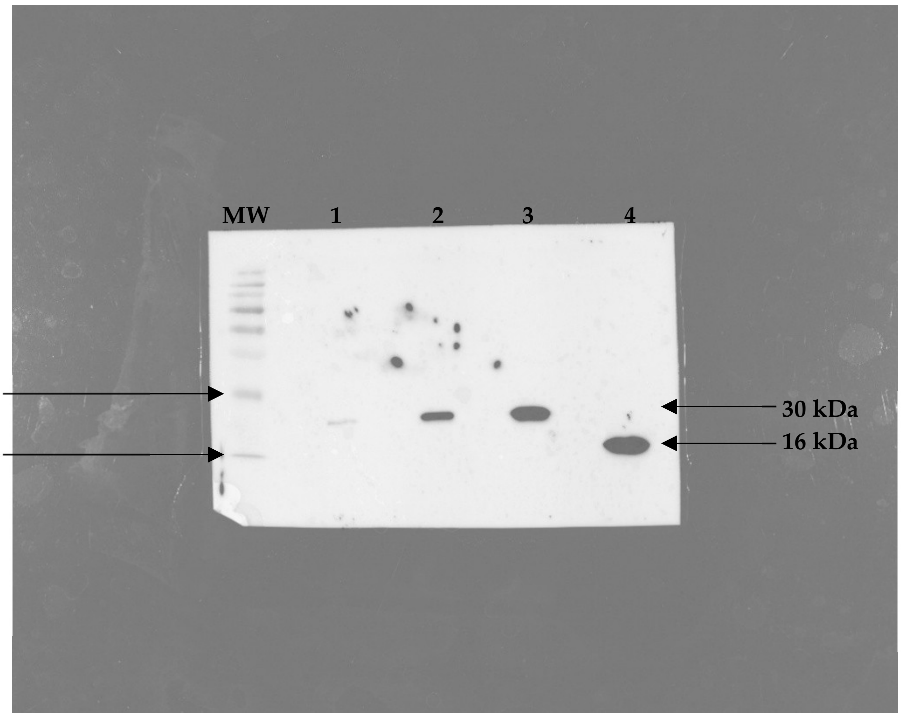

(b) after the merge

Figure 6c:

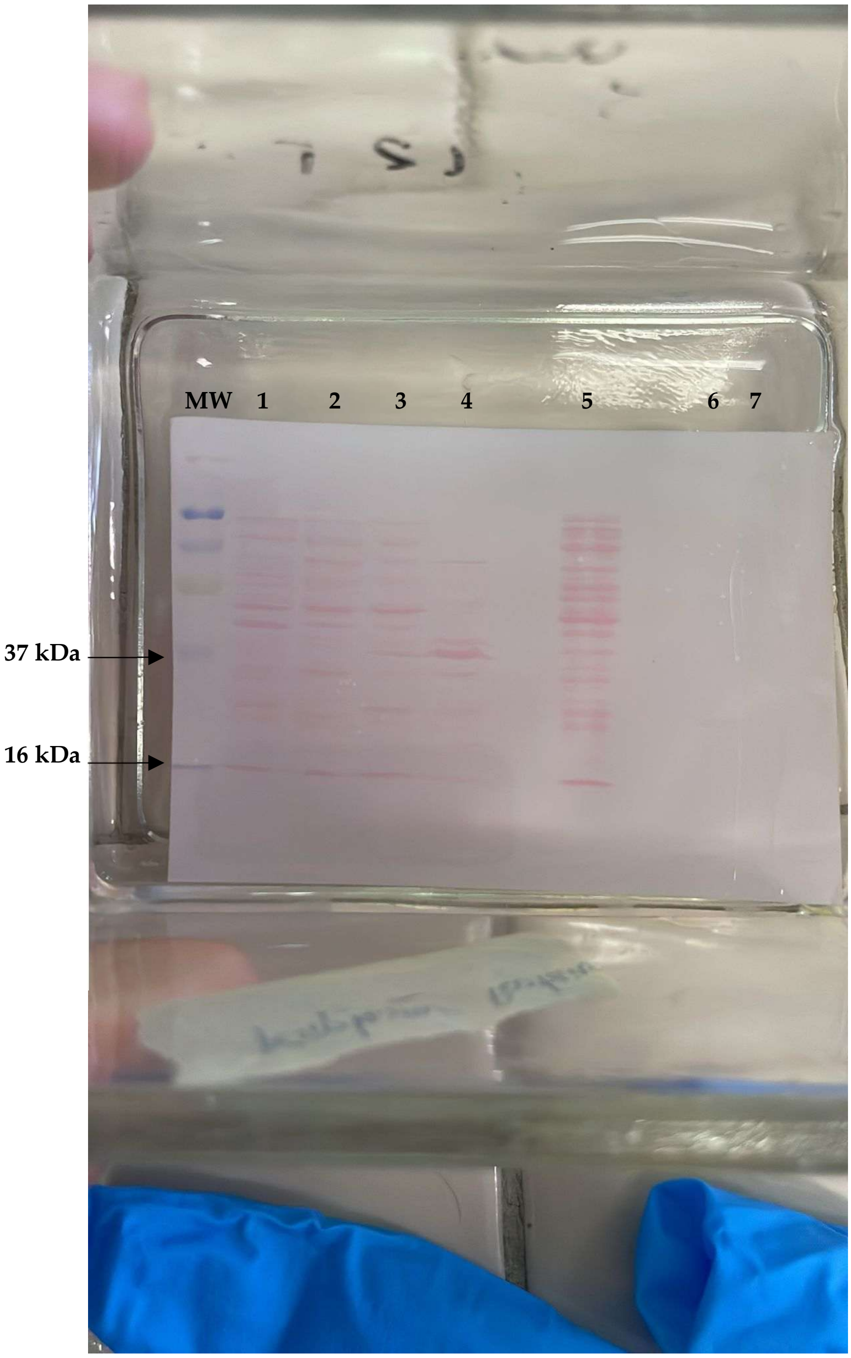

(c)

Figure 6c′ before the merge:

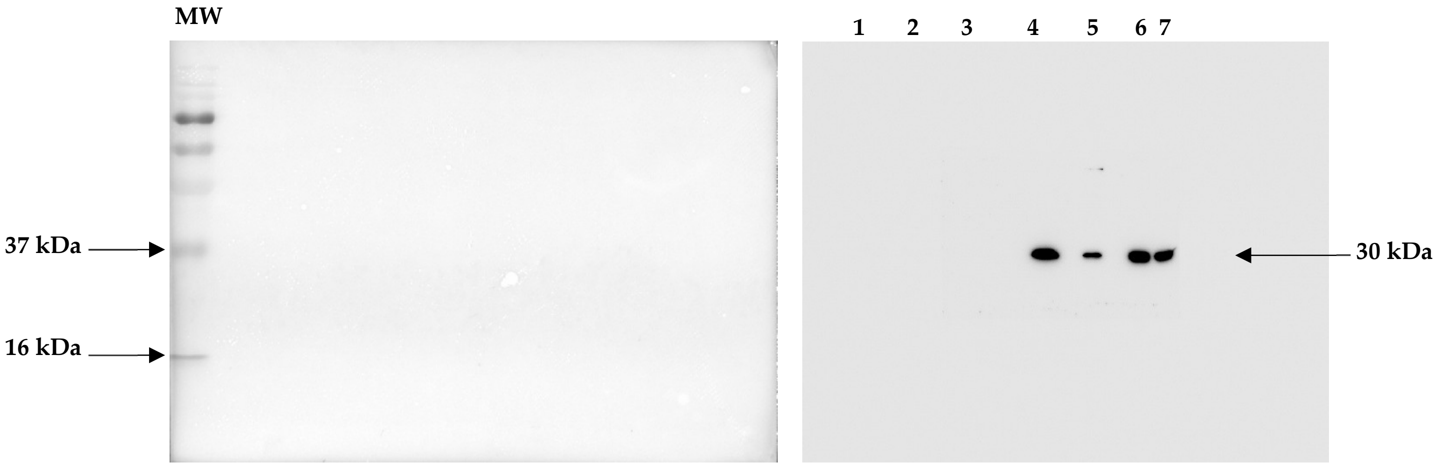

(c′) before the merge

Figure 6c′ after the merge:

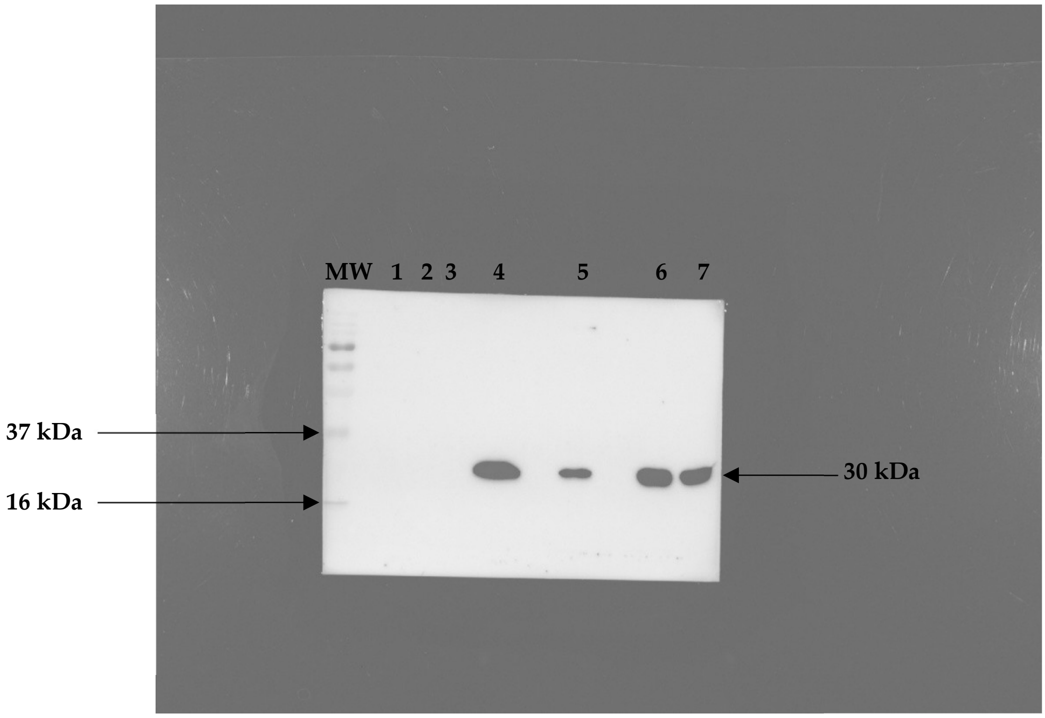

(c′) after the merge

Figure 6d:

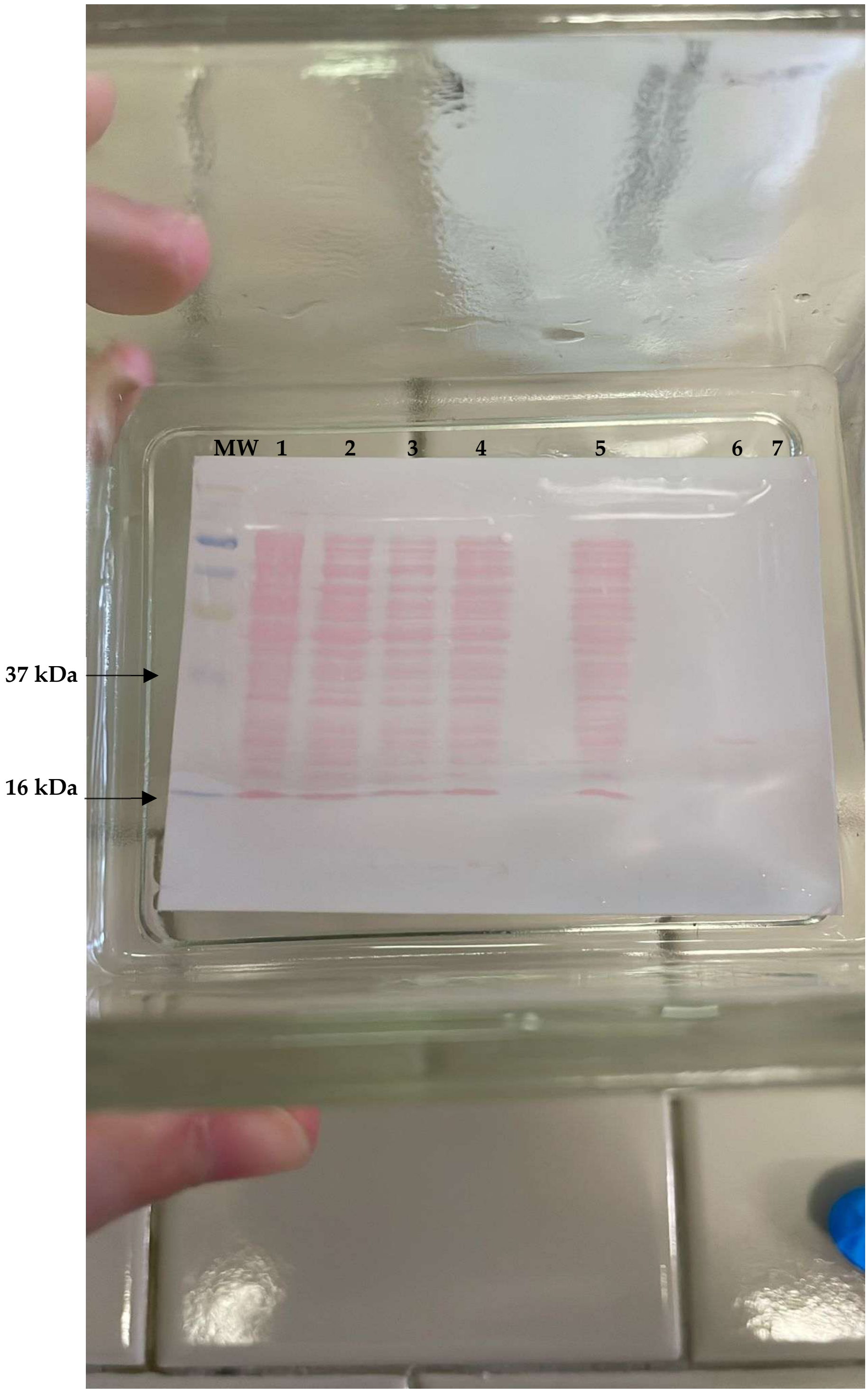

(d)

Figure 6d′ before the merge:

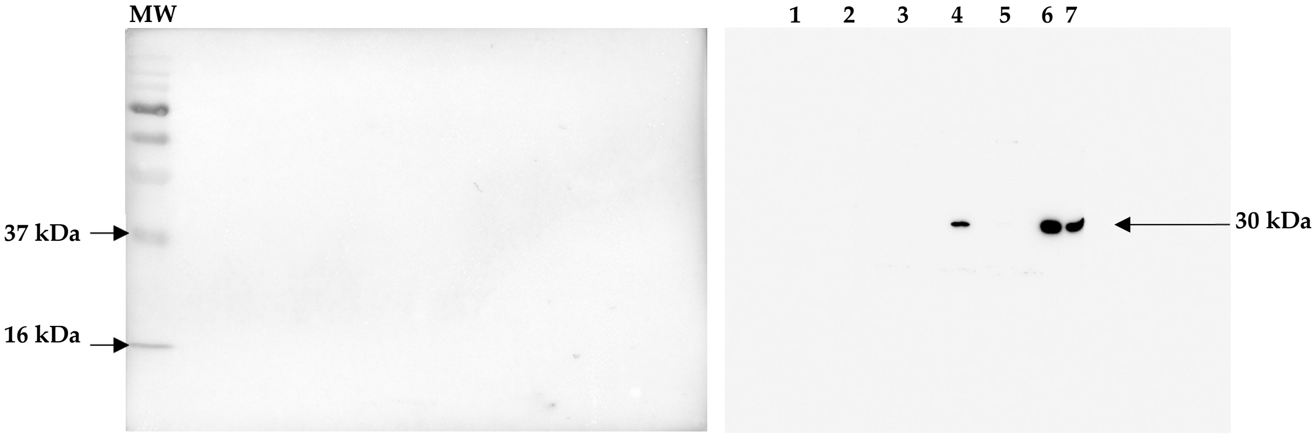

(d′) before the merge

Figure 6d′ after the merge:

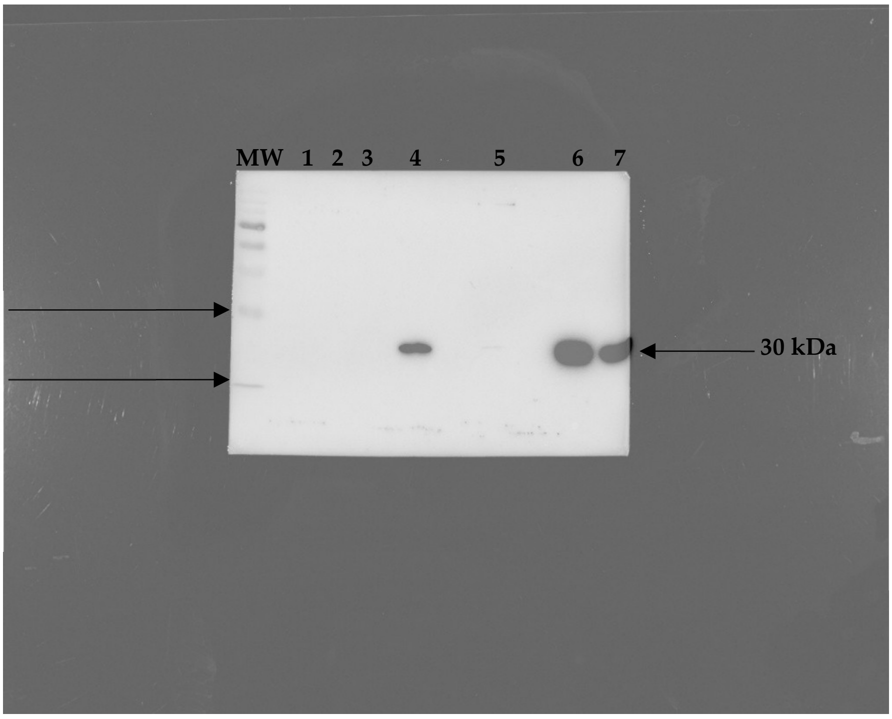

(d′) after

Figure 6. Western blot analysis of Nb25 specificity for recombinant KPC-3 and clinical isolate lysates. (a) SDS-PAGE (16%) of purified proteins showing distinct bands for Nb25 (~16 kDa) and KPC-3 (~30 kDa), confirming purity. (b) Western blot demonstrated concentration-dependent binding of Nb25 to denatured KPC-3. Lanes 1–3: 125, 250, 500 ng KPC-3; lane 4: 500 ng Nb25 alone (control). (c) Ponceau S staining of periplasmic extracts (20 µg/lane) from clinical bacterial strains. Lane 1: *E. coli* (NDM-1); lane 2: *K. pneumoniae* (IMP-1, SHV-12); lane 3: *K. pneumoniae* (OXA-1, CTX-M-15, OXA-48); lane 4: *K. pneumoniae* (KPC-2); lane 5: *E. coli* (KPC-3 + others); lane 6: recombinant *E. coli* BL21 expressing KPC-3; lane 7: purified KPC-3. (c′) Corresponding Western blot probed with Nb25 (2 µg/mL) and anti-HA antibody (1:20,000), followed by HRP-conjugated secondary antibody (1:7,000). (d) Ponceau S staining of cytoplasmic extracts (25 µg/lane) from the same strains.

(d′) Corresponding Western blot showing Nb25 binding. **A prestained protein ladder (SHARPMASS™ VII, 6.5–270 kDa; Cat. No. EPS026500) was used as a molecular weight reference.**

Notes:

- Using the ChemiDoc™ MP Imaging System (Bio-Rad), the molecular weight (MW) ladder is captured separately under Colorimetric (pre-exposure) settings to ensure accurate visualization, while the protein bands are captured under chemiluminescent exposure after antibody probing. These two images are then merged within the ChemiDoc software to generate the final figure. In this document, we have included the assembled (after merge) image, which shows both MW ladder and all protein bands together on the same page with the original black background, reflecting the unaltered raw data and confirming that all samples were run on the same gel.
- All original uncropped images, including the separate MW and protein captures, are provided. No lanes were removed, rearranged, or duplicated.
